# Synthesis, Characterization, and Photocatalytic Activities of Graphene Oxide/metal Oxides Nanocomposites: A Review

**DOI:** 10.3389/fchem.2021.752276

**Published:** 2021-09-21

**Authors:** Hayfa Alajilani Abraheem Jamjoum, Khalid Umar, Rohana Adnan, Mohd. R. Razali, Mohamad Nasir Mohamad Ibrahim

**Affiliations:** ^1^ School of Chemical Sciences, Universiti Sains Malaysia, Pulau Pinang, Malaysia; ^2^ Department of Chemistry, Faculty of Science, University of Sabratha, Sabratha, Libya

**Keywords:** graphene oxide, metal oxide, photocatalyst, zinc oxide nanocomposite, titanium oxide nanocomposite

## Abstract

Sustainable water processing techniques have been extensively investigated and are capable of improving water quality. Among the techniques, photocatalytic technology has shown great potential in recent years as a low cost, environmentally friendly and sustainable technology. However, the major challenge in the industrial development of photocatalyst technology is to develop an ideal photocatalyst which must have high photocatalytic activity, a large specific surface area, harvest sunlight and shows recyclability. Keeping these views, the present review highlighted the synthesis approaches of graphene/metal oxide nanocomposite, characterization techniques and their prominent applications in photocatalysis. Various parameters such as photocatalyst loading, structure of photocatalyst, temperature, pH, effect of oxidizing species and wavelength of light were addressed which could affect the rate of degradation. Moreover, the formation of intermediates during photo-oxidation of organic pollutants using these photocatalysts is also discussed. The analysis concluded with a synopsis of the importance of graphene-based materials in pollutant removal. Finally, a brief overview of the problems and future approaches in the field is also presented.

## Introduction

Clean and safe drinking water is essential for human health. Human health requires clean water, free of poisonous and carcinogenic compounds and dangerous microorganisms. Rapid industrialization and increase in the rate of population have been a matter of urgent need for authorities, and civil society all over the world. The United Nation (UN) Water Development Report 2020 estimates the lack of pure drinking water for over 748 million people and the water used by the manufacturing sectors would grow by a stunning 400% by 2050 (Ahmed and Haider, 2018; WWAP, 2018). Drinkable water is a key problem, particularly in regions that do not manage the eradication of toxic metal ions and industrial waste. As a result, a large number of harmful organic and inorganic compounds in the water bodies pose a major threat to human health (Escherichia et al., 2020). To address such kinds of issues there is a critical need to develop the wastewater treatment technique. Previously, major works were devoted to upgrading the natural aerobic techniques, coagulation, precipitation, diagnosis of membranes, photocatalysis, Fenton reactions, microbial fuel cells and adsorptions. Though, various restrictions–the difficulty of procedures and their time-consuming nature, increased operations costs and chemical agents utilized, aggregate loam generation and separation challenges were also hindered their prospective application (Raizada et al., 2020). In contrast to all of these constraints, advanced oxidation techniques are regarded to be among the best suitable methods for effective wastewater treatment based on photocatalytic heterogeneous and homogeneous oxidation of multiple organic contaminants. Photocatalytic reaction is a reaction that occurs under light and in the presence of a photocatalyst. This technology has several advantages, including protection of the environment, full pollutant degradation and no secondary pollution (Zhang et al., 2019). A wide range of potential photocatalysts restrict their use only with UV radiation and, thus, substantially increase their costs, particularly because solar light constitutes only ∼5% UV radiation; therefore, need to provide a light energy by an external source (Moma and Baloyi, 2019; Umar et al., 2019). Several metal oxides have been examined as a heterogeneous photocatalyst for wastewater treatment (Yaqoob, Noor, et al., 2020). Few examples are iron (III) oxide, zinc oxide (ZnO), niobium pentoxide, titanium oxide (TiO_2_), tungsten trioxide, vanadium oxide and zirconia (Raizada et al., 2017).

Among all, ZnO and TiO_2_ are the most prominent material for their use as photocatalysts to treat the wastewater. ZnO has shown considerable potential in recent years due to its unique features such as its reasonable cost, strong oxidation capacity, extraordinary photosensitivity, bio consistency, acceptable photocatalytic performance, high chemical stability (Mullani et al., 2020), outstanding pyroelectric, and piezoelectric characteristics (Lee et al., 2016). Unlike the bulk materials, ZnO have a high definite surface area (i.e., very acute areas) which is having a critical responsibility in the generation of photogenerated charge carriers and reactive oxygen species (ROS) such as hydroxyl radicals which enable pollutant adsorption and mineralization when treated with UV light. Moreover, the comparatively wide bandgap (around 3.37 eV) requires UV radiation for the reaction to take place and the fast recombination and low resistance to corrosion prevent the practical application of electron-hole pairs. To date, ZnO performance has been improved using numerous strategies including metal loading, ion doping, connection with semiconductor, composite loading, and improved structural design etc (Dhodamani et al., 2020). The use of carbon based material to prepare visible light active photocatalysts or prolong the life of photo-generated electron-hole pairs is the most promising among the others. Although, ZnO has been combined with conventional carbon-based materials, graphene and its derivatives are one of the most promising options for this purpose (Chauhan et al., 2019). These materials show exceptional surface properties as well as outstanding electrical, thermal, chemical and adsorption characteristics. Graphene oxide (GO) is a good option for ZnO-based integrating photocatalysts, due to the surface functionality of hydroxyl/carboxyl groups and its potential for charge separation properties (Gebreslassie et al., 2013; Umar et al., 2015; Serrà et al., 2019).

GO-based ZnO nanoparticles have recently become increasingly common in photocatalytic applications due to their superior physicochemical and photo-electrical characteristics. As the *p*- conjugation structure displays great electronic mobility, the structural properties of GO-based nanocatalysts may increase the photocatalytic performance of ZnO considerably. The GO/ZnO composite increases the separation of electron-hole pairs on the surface of ZnO and increases the harvesting the light energy ability in the visible region. In addition, they offer high efficiency because of the high specific surface, numerous active sites, and strong supporting properties (Haseen et al., 2020; Kumar et al., 2018; Yaqoob et al., 2020).

Similarly, TiO_2_ is also one of the most promising materials as a photocatalyst. Several investigations on the photocatalytic ability of TiO_2_ have been reported for different uses, including organic pollutants degradation and air cleaning etc. (Mir et al., 2012; Umar et al., 2012; Umar et al., 2016; Tobaldi et al., 2021). However, overall, the photocatalysts have some disadvantages such as cost-effectiveness, low efficiency, high recombination and requires UV-radiation. TiO_2_ is widely utilized in wastewater treatment study as a photocatalyst due to its 1) excellent stability, 2) low price and low toxicity and 3) improved photocatalytic activity compared to other semiconductor materials (Mir et al., 2013; Umar et al., 2013; Malik et al., 2014; Bhanvase et al., 2017; Hunge et al., 2021). Therefore, photocatalytic activity is restricted to the visible Sun spectrum region. The rate of electron and hole recombination in TiO_2_ is also greater. The preparation of TiO_2_ composite-based photocatalysts with graphene derivatives is a way to resolve these issues. The electron mobility in case of graphene is greater, which improves the electron transportation and reduces the recombination of electron and hole. Moreover, the usage of graphene–TiO_2_ composite, TiO_2_ agglomeration may help prevent greater surface area and more active sites for pollutant degradation. It also increases the pollutant degradation rates by using a lower quantity of graphene–TiO_2_ composite photocatalyst. Graphene has high heat conductivity, improved load carrier’s mobility and a superior mechanical strength (Yaqoob et al., 2021) and also a potential material for enhancing TiO_2_ photoactivities through these electrical and photonic capabilities (Dhodamani et al., 2020). The photoactivity of graphene–TiO_2_, as a result of higher visible light absorption and high affinity with other organic materials, is also improved (Jawaid et al., 2019). This review focuses on the characteristics, synthesis, and their important role in the photo-degradation of ZnO and TiO_2_–GO nanocomposites. In this article, the characteristics of ZnO, TiO_2_ and GO, methodologies for GO/metal oxide nanocomposites synthesis, the photocatalytic mechanism for degradation, factors effecting the efficiencies of the photocatalyst, existing difficulties are discussed and recommendations for future works are proposed.

### Significance of Graphene Derivatives for Photocatalysis

**FIGURE 1:**
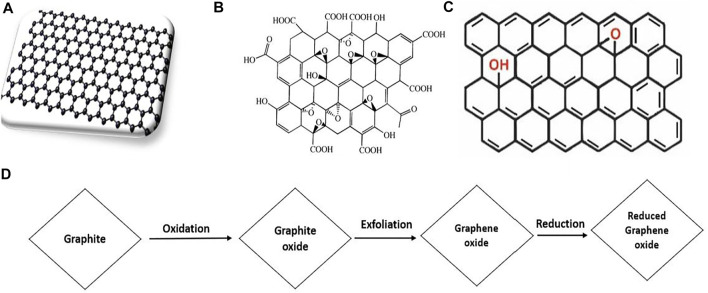
Structure of **(A)** single layer graphene, **(B)** graphene oxide (GO) **(C)** reduced graphene oxide (rGO) and **(D)** the synthesis route of rGO through GO reduction (Adapted from Yaqoob et al. (2020) with MDPI permission).

In general, graphite, diamond, and amorphous forms of carbon are classified, depending on the atomic structure and characteristics of carbon atoms. Graphene has 2D carbon with a nano-thick atomic sheet. The nanomaterials are directly concerned to such a unique substance since graphene is one of its fundamental constituents (Hussain et al., 2019). Graphene has also become a prominent material in a wide array of industries and applications, including energy conversion and storage beside its photocatalytic application (Ahmad et al., 2019; Dhodamani et al., 2019). As demonstrated in Figures 1A–D, graphene exists in many forms and they are generally used to manufacture heterogeneity in a range of applications. It also provide significant potential for generating redox vibrating media and water purification catalysts because of their vast surface and catalytic, optical, and electric characteristics (Shandilya et al., 2018). The very porous nature and widening surface of carbon-based nanoparticles also has strong adsorption characteristics, providing various active facilities for wastewater treatment (Singh et al., 2020).

GO is a relatively having extensive applications, formed by exfolation of graphite oxide. The first trial to synthesize GO was performed by Benjamin C. Brodie (1859), a chemist at Oxford University, and L. Staudenmaier in 1898 (Gao, 2012). Hummers et al. subsequently proceeded researching the substance using a potassium and nitric acid combination in graphite for the chemical reaction (Chen et al., 2015; Staudenmaier, 1898). Later, G. Offeman and Hummers tried, but with minor alterations, as seen in Figure 1D. Initially, 100 g graphite powder in sulfuric acid is combined with 50 g sodium nitrate. The mixture is then chilled on an ice bath to verify the presence of contaminants. Thirdly, in an agitated mixture of 300 g of potassium permanganate, graphite begins to oxidize, with a very thorough and tiny quantity of potassium permanganate to remain under 20°C in the suspension temperature. Fourth, with a tiny quantity of gas developed after 20 min of mixing, the combination suspension turns to a brownish-grey paste. Fifth, the mixture is supposed to be continuous for 30 min after which deionized water gently is added to the paste that generates a strong sparkling wave at 98°C. Sixth, the diluted paste holds for 15 min when brown has turned. Seventhly, more deionized water is added to enhance dilution, adding H_2_O_2_ to remove manganese dioxide. The addition of peroxide leads to a vivid yellow suspension. Eighth, the overflowers are filtered in suspension, and the yellow-brown filter has to be rinsed two or three times. Ninth, the heated powder of the filtered goods is 40°C. Finally, there is a single atomic sheet of GO (Ramachandran et al., 2015; Ortiz Balbuena et al., 2016).

Moreover, based on the surface functionalization in the development of graphene semi-continuous composites with tunable textural and surface chemical features, carboxylic, hydroxyl and epoxy groups combine with valuable functionality compounds such as polymeric materials and metal oxides and accountable to use in different applications (Vadivel et al., 2019). Furthermore, graphene-based nanocomposites thus offer an excellent field of study on water remediation and decontamination systems. Additionally, commercial graphene is very expensive to use as a photocatalyst, therefore the synthesis of graphene by using waste material via a hummer method could be the best option. Several efforts are ongoing on utilization of agro-waste material, biomass, paper etc. to prepare the GO, which can be further doped with metal oxide to use as a photocatalyst (Safian et al., 2020). This step can make the technique more reliable and cost effective.

### Graphene Oxide/metal Oxide Nanocomposite Synthesis Methods

Due to their nano-size and good optoelectronic characteristics, the nanocomposites GO/ZnO and GO/TiO_2_ received considerable interest in the area of the photocatalysis. The lowering of recombination process and improved the resistance to photo-corrosion for the ZnO and TiO_2_ photo catalyst will be of great concern. The integration of ZnO and TiO_2_ nanoparticles into GO surface resulting in high stability and preventing graphene-based layers from being joined due to strong van der Waals pressures that exist among graphene-based layers. In material science, synthesis of new, more efficient nanocomposites based on graphene oxide/metal oxide is becoming more and more important and some regularly used techniques are listed as follows.

### Sol–Gel Method

**FIGURE 2:**
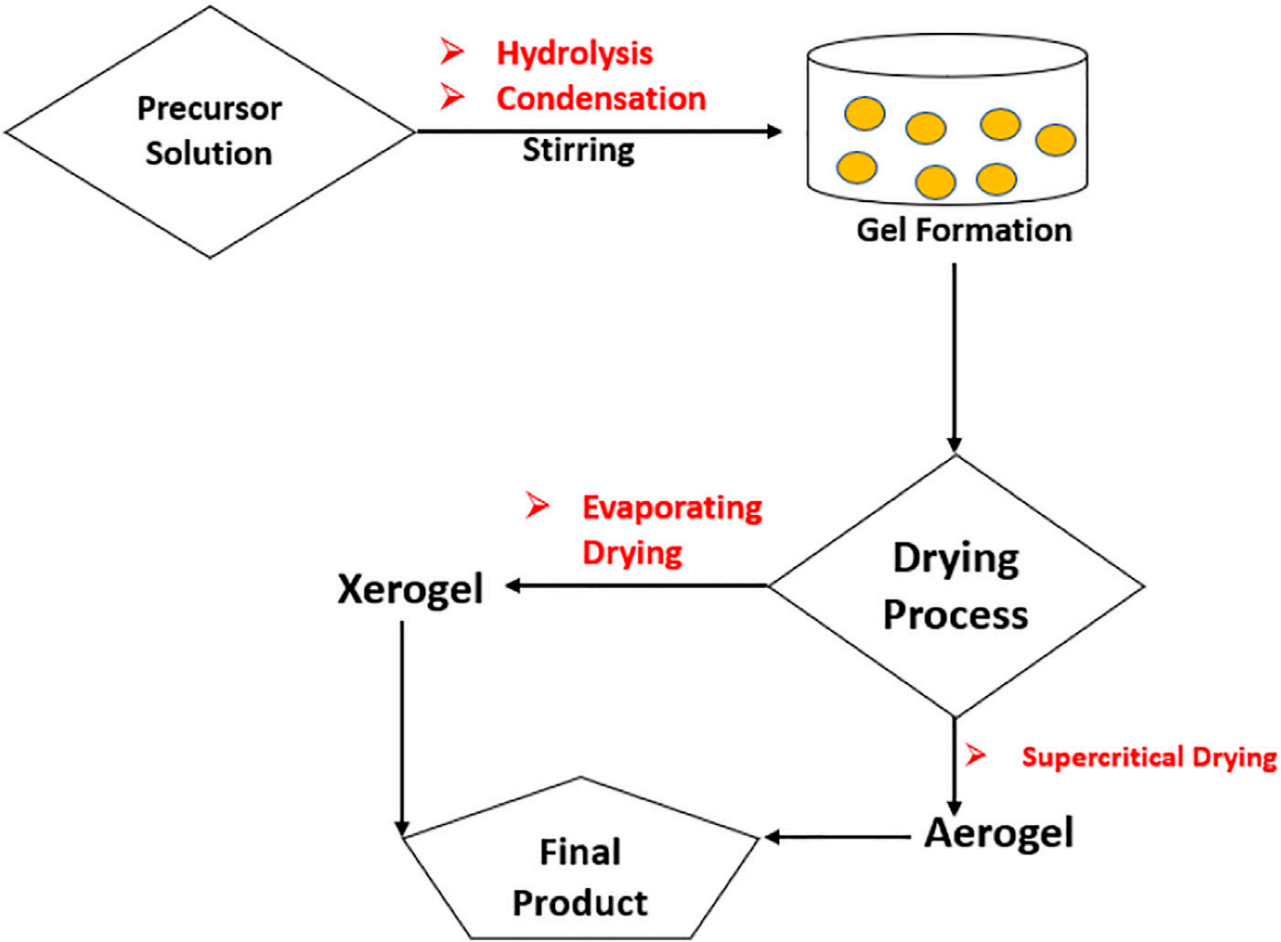
Graphic presentation of sol-gel technique (Reproduced from Yaqoob et al., 2020 with MDPI permission).

Sol-gel is one of the most promising approaches to produce composite, which was originally used in late 1980s, for the synthesis of nanocomposites. The sol-gel technique is a multi-step procedure based on the metal precursors sequentially hydrolyzed for a metal hydroxide resolution followed by an instantaneous condensation to create a 3D gel, as illustrated in Figure 2.

**FIGURE 3:**
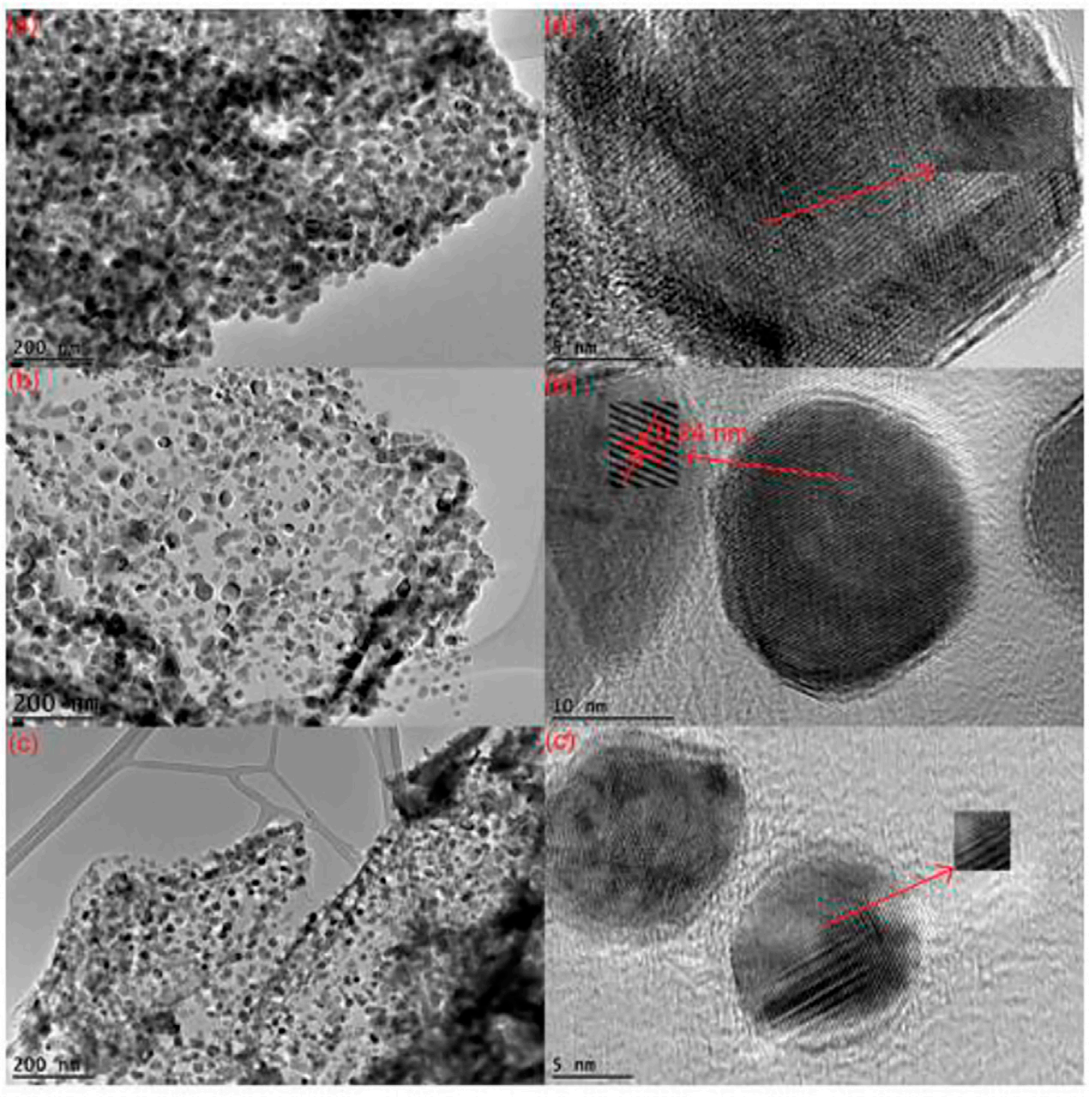
TEM and HRTEM images of ZnO – rGO nanocomposites via sol-gel method at **(A)** low, **(B)** mid and **(C)** high rGO concentration (reproduced with permission from Azarang et al. (2014); copyright ^©^ 2020, AIP).

The gel produced is dried, which leads to aerogel or xerogel and the desired product being formed afterwards. Aquatic or non-aquatic solvents are utilized, and results are more porous than other nanocomposites and have a larger area. Graphene materials are suitable precursors to the sol-gel approach because of their strong dispersive capacity to react with covalent aqueous or non-watery chemicals and functions. During the last several decades, ZnO-rGO nanocomposites have been developed using sol-gel methods by including nanoparticles of ZnO on the surface of rGO sheets. One study reported by Azarang et al. on the preparation of ZnO-rGO using sol-gel method to investigate the effect of rGO concentration on the optical properties of the nanocomposites. Figure 3 shows the TEM and HRTEM images of the nanocomposites at different rGO loading. The images reveal that the ZnO NPs are decorated and dispersed on the RGO sheets (Azarang et al., 2014). The major benefit of the sol–gel method is that the functional groups on rGO surfaces offer reactive and anchoring sites for the nucleation and growth of NPs. As a result, the metal oxide nanostructures will be chemically bonded to the GO/RGO surfaces.

### Solvothermal and Hydrothermal Method

Solvothermal and hydrothermal methods played an important role in various scientific fields either for commercial research purposes or basic research. These approaches are favorable as the process is simple, efficient and low-cost. The methods are also environmental friendly as there are no hazardous organic solvents being used. Usually, water or ethanol was used as the solvent and the nanoparticles are formed under a high temperature and pressure for a given time. Using this one-pot method, the metal oxide can be attached and/or wrapped to the rGO sheets simultaneously with the reduction of graphene oxide (Alves et al., 2021). Last few years, these methods developed aggressively, especially in case of the augmentation of nanocrystalline. For a closed system, chemical reaction takes place between single or different precursors in solution like water at any greater temperature than their boiling point. The solvent investigated from its chemical and physical aspects which are dielectric constant, density, polarity, and also interaction between additives and reactants itself.

Marlinda et al. pursued the study of hydrothermal methods for the preparation of ZnO/rGO composites. In basic procedure, graphene solution (20 ml) was produced by Hummers’ method and Zn (OH)_2_ solution (2 ml) obtained from Zn (CH_3_COO)_2_ solution and NaOH (1 g) was subjected to liquefaction in 25% NH_3_.H_2_O (25 ml) respectively. The mixture was added slowly in presence of NaOH solution (0.1 M) for increasing the pH until ∼10 while the solution was kept stirred in an oil bath until dark brownish liquid produced at temperature 60 ± 2°C. Later, the mixture was being exposed into treatment involving hydrothermal methods for a day (24 h) at 180°C temperature. The generation of the end product was ended by washing it with ethanol, deionized water and lastly, dried up into an oven at 60°C temperature (Marlinda et al., 2012). Saravanakumar et al. prepared the ZnO/graphene nanocomposites through a modest temperature range in hydrothermal technique (Saravanakumar et al., 2013). The procedure started by 0.45 g of GO dissolved in 50 ml of deionized water and put inside the sonicated bath for a period time of 30 min and pH solution tuned by adding KOH solution drop wisely. Next, 0.5 M of zinc nitrate hexahydrate was added and stirred for about 15 min. The pH increased using an ammonia solution that amplified alteration speed of Zn (OH)_2_ solution. After 30 min stirring, the solution was then transported to a glass container and wrapped definitely with a Teflon lid, and kept the container in the oven (hot air) for 10 h at distinct temperatures which are 98°C and 80°C. The final product was then cooled until achieved 25°C temperature. The cooled sample was washed by using distilled water and ethanol. Ahmad et al. reported that the ZnO/graphene-Ag nanocomposites fabrication used simple solvothermal technique for organic dyes degradation (M. Ahmad et al., 2013). In previous procedure, 10 wt% of purified GO undergo liquefaction in ethylene glycol (80 ml) through ultrasonication for 2 h was done and followed by centrifugation in order to get yellowish brown suspension. Different weight percent of silver acetate, 0 wt%, 2 wt% and 4 wt% together with 80 mg of zinc acetate were liquefied in 80 ml of ethylene glycol pursued with yellowish brown dispersion addition under continuous stirring (magnetic rod). Next, 20 mg of NaOH was mixed into deionized water (20 ml) and the solution then combined into a reaction mixture with magnetic stirred for 60 min. The mixture will become homogeneous, and it was quickly transferred into stainless steel lined with a Teflon container. The mixture was then autoclaves for overnight (24 h) at 60°C. Lastly, the prepared nanocomposites were washed and dried through the oven.

### Direct Growth of Metal Oxide Nanoparticles on the Surface of Graphene Oxide

**FIGURE 4:**
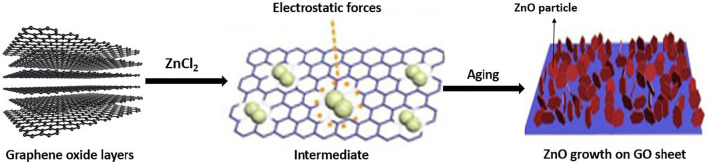
The ZnO nanoparticles growth on the surface of GO layers (Reproduced from Yaqoob et al. (2020) with MDPI permission).

ZnO and TiO_2_/GO nanocomposite characteristically synthesized in optimized ratio. First, Zn^2+^ ions had been deposited evenly on the surface of GO and then turned it into ZnO NPs with the addition of NaOH and NaBH_4_ at 150°C. ZnO/graphene then produced at the end of reaction after graphene oxide reduced into graphene. Generally, ZnO NPs have grown into a range, 10–20 nm size with distribution of exquisite particle physical size on the surface of graphene layers (Li and Cao, 2011). Chang et al. had reported the method for producing ZnO/graphene nanocomposite by using an easy *in-situ* method (Chang et al., 2011). The procedure was started by mixing ZnO quantum dots into graphene in a fixed calculated ratio in order to earn a mixture of ZnO and graphene. Then, the mixture was cast with different substrates e.g., polyethylene terephthalate, quartz, glass and SiO_2_/n-Si to generate ZnO/graphene thin films. Next, the films now were hardening by evaporating it at temperature 100°C and captivated inside zinc nitrate solution to *in situ* escalate ZnO quantum rods into ZnO nanorods where these nanorods used to make ZnO/graphene nanocomposites. These reactions aimed to enhance the substrate’s adhesion. Lastly, nanocomposite was washed several times to remove the impurities from ZnO nanorods. Feng et al. reported in his research about exploration of new easy method to synthesis ZnO nanosphere/rGO nanocomposites through a hydrothermal supported *in-situ* gelatin method and also explained the ZnO dispersion effect on rGO (Feng et al., 2014). According to the conclusion, the ZnO/rGO nanocomposite was prepared by using Zn (Ac)_2_. 2H_2_O and GO at different ratios, which they were distinctly added into the gelatin mixture. This reaction was done under mixing and stirring continuously at room temperature. At the same time, NH_3_.H_2_O of 25 wt% were put drop wisely into mixture that was obtained before until the mixture achieved pH 9.0–10.0 proceed by stirring with magnetic rod continuously for fixed 30 min. After that, the obtained product was conveyed into Teflon lined (25 ml) with stainless steel-based autoclaves for the same amount of time reaction as in ZnO synthesis. Last step, the end product obtained was washed with water, then dried out in vacuum at temperature 80°C. RGO compounds had excellent electron transportation and improved well surface area that contributed good effects to improve the nanocomposite’s photocatalytic reaction. Sahatiya and Badhulika also reported about an easy as one-step method for single graphene *in-situ* preparation which doped with zinc oxide (GO-ZnO) nanocomposite through using electrospinning (Sahatiya and Badhulika, 2015). However, the general systematic proposed mechanism by *in-situ* growth of ZnO particles on the surface of graphene is present in Figure 4.

### Solution Mixing Method

Solution mixing is a direct and efficient method that has been extensively used to prepare graphene/metal oxide composites. This method can be conducted at a low temperature. Besides, it promotes faster de-aggregation and dispersion of reinforcement material and produces composites with uniform structure. Prabu et al. prepared ZnO-rGO composite by a direct solution mixing method. Initially, a spindle-shaped ZnO was prepared by hydrothermal method and the rGO was synthized using Hummer’s method. From the study, they observed the spindle-shaped ZnO were found to be decorated on the partially reduced graphene oxide sheets (Prabhu et al., 2018). In another study they report a study on dumbbell-shape ZnO-rGO nanocomposites. Similar to the previous study, the nanocomposites was prepared by a simple solution casting method. They used different concentration of rGO. In this study, a corresponding amount of rGO was dispersed in 25 ml de-ionized water for 1 h. Then, 100 mg of the dumbbell-shape ZnO was added and continuously stirred for 12 h. After that, the mixture was washed with ethanol and de-ionized water for several time. Finally, the ZnO-rGO was dried overnight in an oven at 80°C. The produced nanocomposites was used as a catalyst for the degradation of organic pollutant (Prabhu et al., 2019).

### Self-Assembly Method

Self-assembly is an efficient process in to assemble micro or nanomaterials into some specific ordered structure or pattern. This was achieved by the interactions among the materials themselves. Previously, Du et al. fabricated ZnO-rGO hybrid films via self-assembly method for use in supercapacitor application. In their study, they first synthesized ZnO nanoflakes and use it as a layers structure spacer. The hybrid film was achieved through ultrasonic mixing and subsequent self-assembly by vacuum-assisted filtration (Du et al., 2019). Another study was conducted to prepare a nanostructured graphene-TiO_2_-porphyrin composite via surfactant-assisted self-assembly for degradation of Rhodamine B. In their work, a graphene-TiO_2_ porphyrin hybrid material was synthesized by CTAB surfactant and graphene-assisted co-assembly of monomeric TCPP molecules with TiO_2_ nanoparticles. They composites with good integration of TiO_2_ particles (15–30 nm diameter) and aggregated porphyrin nanorods (50–60 nm width) and several hundreds of nanometers in length on the graphene surface (La et al., 2017).

### Microwave Irradiation

**FIGURE 5:**
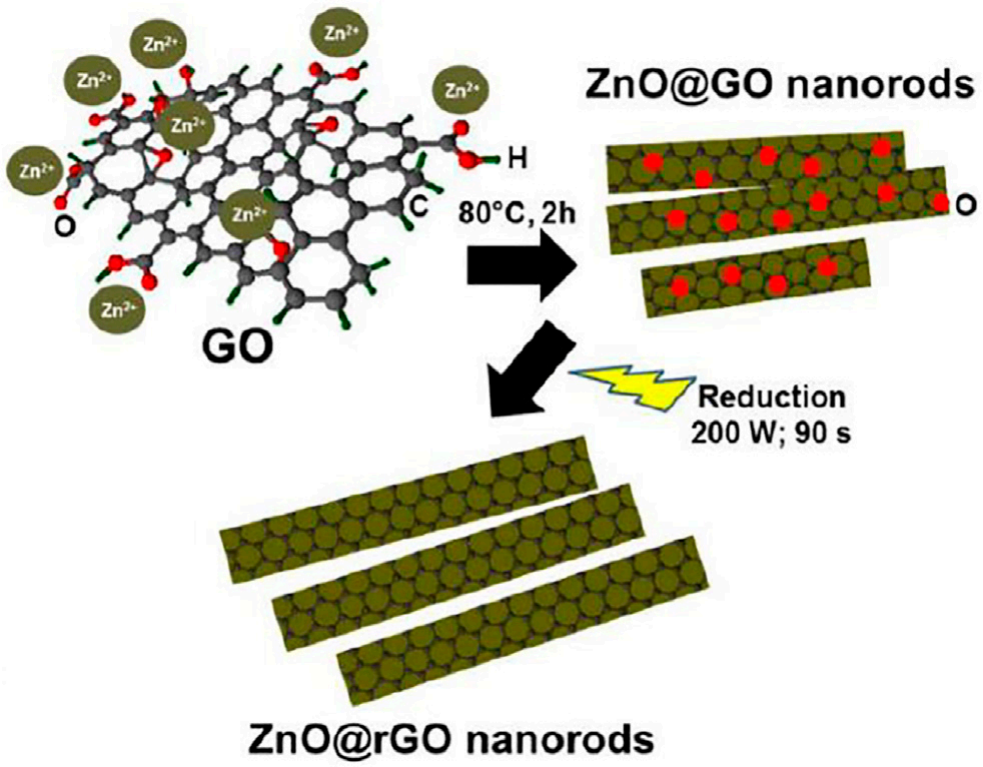
Representation of the ZnO@rGO NR hybrids synthesis via microwave-assisted method. (Reproduced from Jana and Gregory (2020) with WILEY permission).

Microwave irradiation is a rapid method that provides energy for chemical reaction. It generates rapid intense heating in the sample interior and thus reduces reaction times. In one study, rGO incorporated MOF-derived ZnO composites were prepared via the microwave-assisted method assisted reduction of GO in MOF-derived ZnO suspensions. In brief, 16 mg MOF-derived ZnO was added into 20 ml GO solution. The solution was put into a focused microwave system and treated at 150°C with microwave irradiation power of 150 W for 10 min. From this study, they obtained a good combination between ZnO and RGO as the surface of curled RGO nanosheets is packed densely by ZnO particles. The resulting composites were used as photocatalysts for the photocatalytic degradation of methylene blue (Zhu et al., 2017). Jana and Gregory synthesized ZnO-rGO core-shell nanorod hybrids using a microwave-assisted system. The representation of the synthesis is shown in Figure 5. In the first part, partial reduction was attained by chemical reduction under alkaline condition. ZnO was added into a round bottom flask containing NaOH and ethanol. Then, ethanolic solution containing GO was added after the mixture was stirred for 15 min. Next, the solution was refluxed for 2 h at 80°C. The suspension was centrifuged and the solid was washed with ethanol and deionized water before drying in oven at 40°C overnight. Finally, the dried powder was irradiated at 200 W in a reactor under vacuum for 90 s (Jana and Gregory, 2020).

### Ball Milling Method

Ball milling is another interesting approached to prepare graphene/metal oxide composites. This method is known to be environmentally friendly, cost effective and scalable method to produce the composites. During the ball milling process, a powder mixture is placed in a concealed container and undergoes high energy collision from the balls that create localized high pressure. This can be used to produce graphene from the graphite ruptured and can be further mixed with other metal oxide. Yin et al. prepared CIPs/ZnO/Graphene ternary lamelliform hybrid using ball milling method for using as a candidate in the absorption of low frequency microwave. The set up was consist of a ball milling tank (500 ml) and grinding balls made up of stainless steel (5 mm). They fixed the ration of grinding balls to samples at 20:1 at which the mass of the balls is 500 g and the materials are 25 g. Ethanol was used as dispersant in the process. The materials were milled under the rotate speed of 500 rad/min for 12 h, and then the final product was separated from the grinding balls and dried under 60°C for 6 h. The hybrid composites showed an excellent absorption performance due to the external planar anisotropy with flake shape after ball milled (Yin et al., 2019). Additionally, study on ZnO-graphene nanocomposites was conducted by Lonkar et al. They used ball milling method and solution-based hydrothermal method. They found out that the ball milling method produced higher surface area and smaller ZnO particle size compare to the other method. Moreover, this study also reported that the composites from ball milling method have a superior photocatalytic activity than the composites form hydrothermal method (Lonkar et al., 2019).

## Characterization Techniques

Composites preparation of GO/metal oxide is often characterized by several parameters like surface properties, optical activity, size, crystallinity, heat stability, shape, etc. These characteristics are the important informations that is required to develop composites with excellent properties. Herein, the characterization techniques are divided into following types; spectroscopic, microscopic and elemental analysis including crystalline phases analysis using XRD technique. Other characterization on thermal and physical properties of the composites will also be included in the discussion.

### Spectroscopic Analysis

Fourier transform spectroscopy (FTIR) is widely used techniques to identify organic compounds and in some cases, inorganic compounds. It is a rapid and non-destructive method that could detect a range of functional groups and is sensitive to changes in molecular structure. FTIR measures the absorption of infrared radiation by the sample material against wavelength. From there, the infrared absorption bands identify molecular components and structures. This method is well fitted with regard to surface chemicals, surface chemical residues, and identification on the surface of NPs for organic functional groups (e.g., ketones, amines, etc.). In the case of GO/metal oxide composites, FTIR is best fitted with Raman spectroscopy to get the full view of the composites properties.

Another important technique that is commonly employed for GO-metal oxide nanocomposites is ultra-violent spectroscopy (UV-vis). UV-vis measure the amount of UV light absorbs by a substance. It can be used to determine analyte concentrations or the chemical conversion of a component. The range of reflection and absorption is measured so that the nanocomposites visible and ultraviolet optical activity is defined (Aslam et al., 2021). The characteristics of various metal nanoparticles ranging from 2 to 10 nm could be based on light wavelengths of between 300 and 800 nm. Similarly, the bandgap of the synthesized material via UV-Vis’s spectroscopy was calculated by Eq. 1.

$$E_{g}=\frac{hc}{\lambda }$$ (1)

**FIGURE 6:**
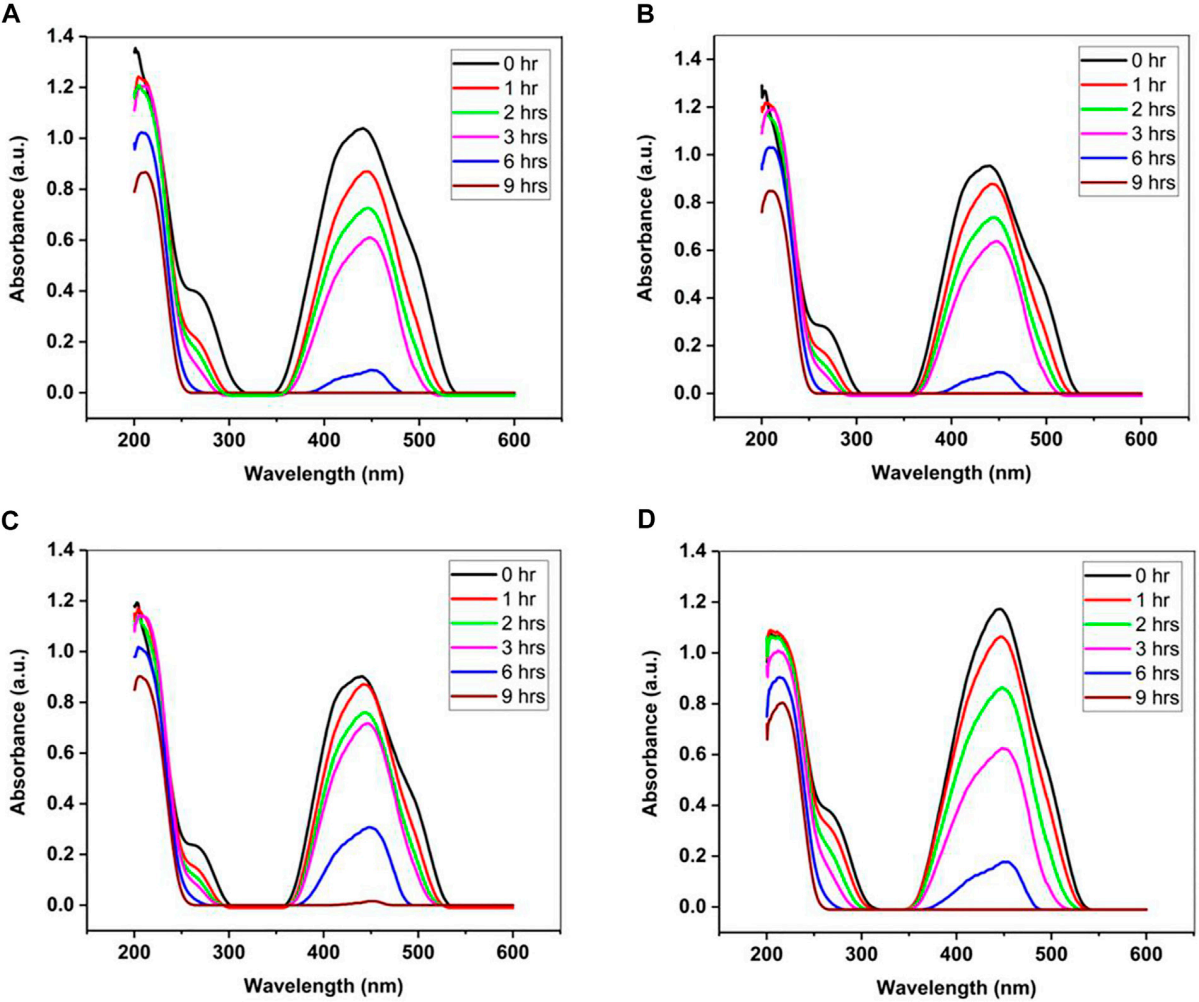
Photocatalytic activity of ZnO-GO nanocomposites at **(A)** 1:10 **(B)** 1:15 **(C)** 1:20 and **(D)** 1:25 ratio. Reproduced from Ahmed and Haider (2021) with MDPI permission.

Where, c = velocity of light (3 × 10^8^ ms^−1^); h = Planck’s constant i.e. 6.626 × 10^−34^ J s; 1 eV = 1.6 × 10^−19^ J and λ = wavelength (Chauhan et al., 2019). UV-vis can be used to study the photocatalytic study of a catalyst as demonstrated by Ahmed and Haider. They synthesized ZnO-GO at four different weight ratios for waste water treatment. From Figure 6, it can be observed that all the samples reached a complete degradation after 9 h irradiation time and the ZnO-GO ratio did not have a significant effect on the photocatalytic performance as they degrade at the same rate (Ahmed and Haider, 2021).

**FIGURE 7:**
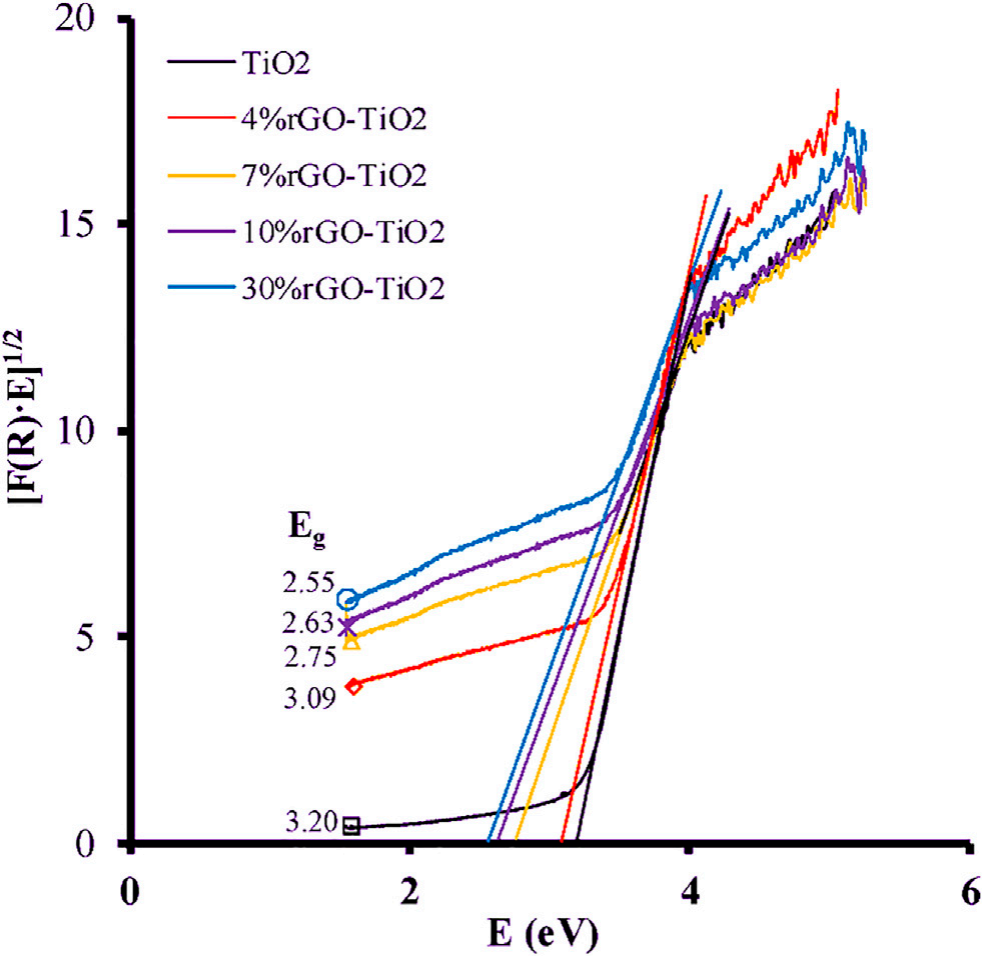
Plots of Kubelka-Munk function and light energy for TiO_2_ and rGO-TiO_2_ composites. Reproduced from Ruidíaz-Martínez et al. (2020) with MDPI permission.

In another study by Ruidíaz-Martinez et al., they used UV-vis technique, to study the electronic structure of the rGO-TiO_2_ composite. The electronic properties were analyzed based on the diffuse reflectance spectra that are used to calculate the band gap energy. The calculation is done according to Kubelka-Munk transformed function as follow:

(F(R)×hV)12=C(hV−Eg) (2)

where n is the constant for the type of optical transition, with values of *n* = 2 for permitted indirect transitions, *n* = 3 for forbidden indirect transitions, *n* = 1/2 for permitted direct transitions, and *n* = 3/2 for forbidden direct transitions. Figure 7 shows plots of the transformed Kubelka-Munk function against the energy of light. The results show that increase amount of GO narrows the band gap energy in composites (Ruidíaz-Martínez et al., 2020).

## Microscopic Analysis

**FIGURE 8:**
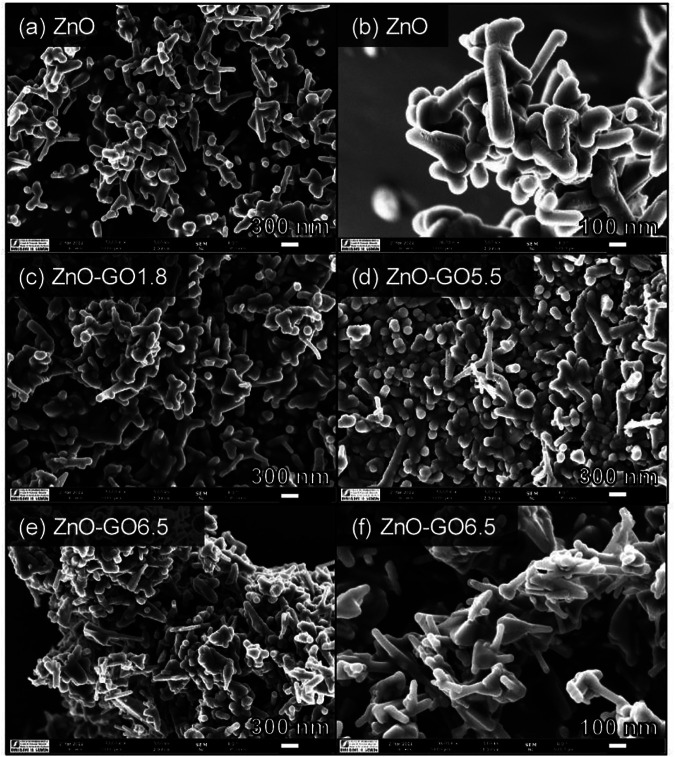
SEM images of ZnO-GO composites at various GO loading; **(A, B)** ZnO, **(C)** ZnO-GO1.8, **(D)** ZnO-GO5.5, and **(E, F)** ZnO-GO6.5. Reproduce from Mirikaram et al. (2021)with MDPI permission.

There are several types of microscopic analysis that frequently used for composites study including scanning electron microscopy (SEM), transmission electron microscopy (TEM). SEM is a technique that may be used to improve the sample’s resolution. The surface morphology, such as determining the exact shape of nanoparticles and topographic analysis, is frequently studied using SEM and TEM methods. The main distinction is that TEM delivers more information about the interior structure and has higher resolution than SEM. The average size of material is also determined using such procedures or approaches. Meanwhile, the AFM method is often employed to collect information on the surface nature, size, shape, topography, roughness, and particle size distribution of nanocomposites (Nabi et al., 2020). The SEM images of ZnO and ZnO-GO at different GO concentration are depicted in Figure 8. It can be seen that, the ZnO structure made up of rod and spherical-like particles. In comparison, the ZnO-GO composites show more aggregated structures than the pure ZnO. From Figures 8C–F, there are no present of GO sheets uncoated with ZnO, indicating a good assembly between ZnO and GO phases for all the composites prepared (Mirikaram et al., 2021).

### Elemental Analysis

**FIGURE 9:**
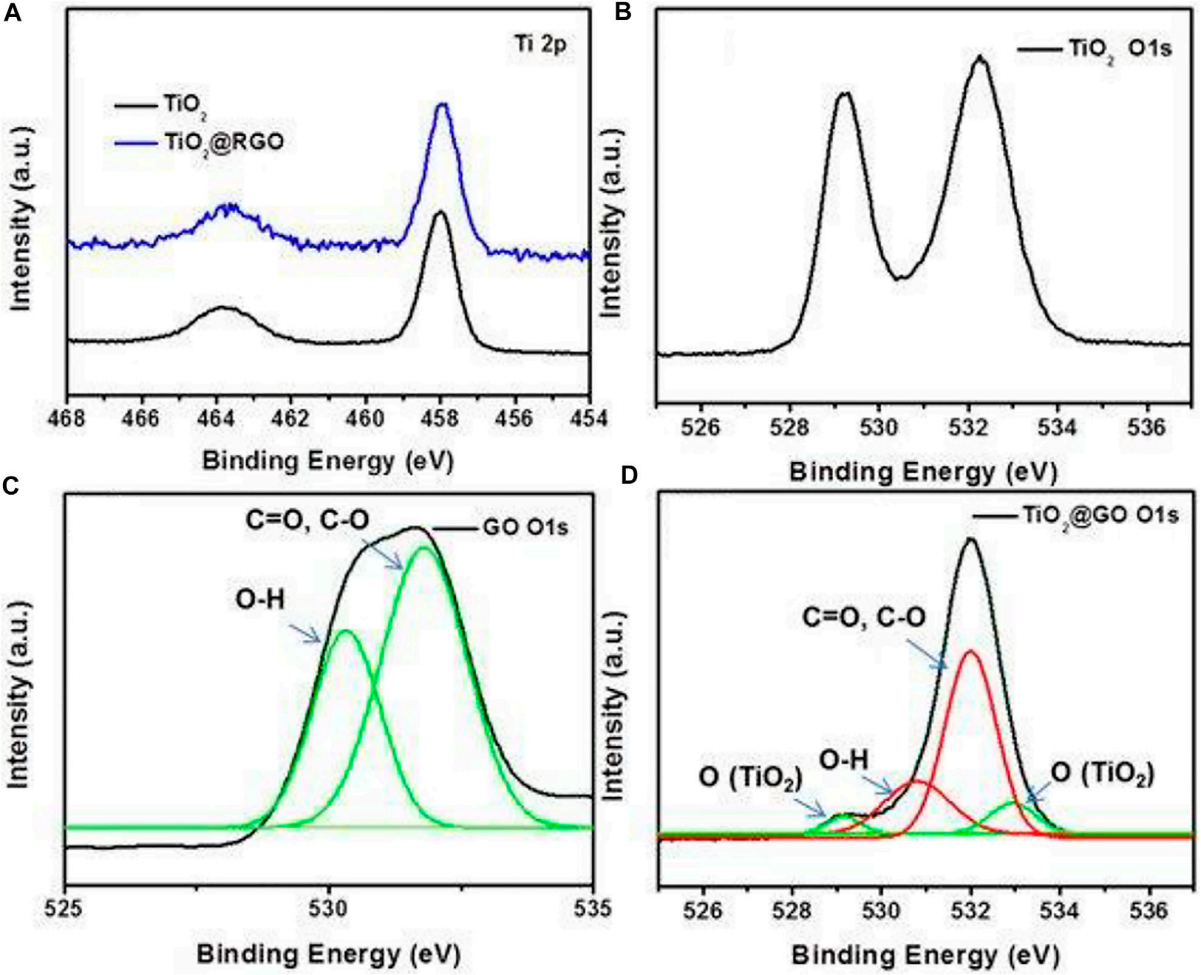
XPS spectra of **(A)** Ti 2p for TiO_2_ and TiO_2_-GO, XPS spectra of O 1s for **(B)** TiO_2_, **(C)** GO and **(D)** TiO_2_-GO. Reproduced from Shi et al., 2021 with MDPI permission.

Elemental analysis can be performed using energy dispersive analysis of X-ray (EDX), X-ray photoelectron spectroscopy (XPS) and X-ray diffraction (XRD). XRD is used to examine nanocomposites’ crystalline phase and is distinguished by diffraction patterns for a crystal structure. X-rays may penetrate nanoparticles in order to generate the particular pattern of diffraction (Wang et al., 2015). In addition, Debye–Scherrer formula and Bragg Law Equations have been utilized to compute the mean crystallite size and d-spacing of the produced material by means of XRD examination using Eqs. 3, 4 as shown below;

$$D=\frac{K\lambda }{\beta Cos\theta }$$ (3)

$$d=\frac{\lambda }{2Sin\theta }$$ (4)

where, *K =* Scherrer constant, *Β* = full width at half-maximum (FWHM), *λ* = wavelength of the X-ray, D = particle size and *θ* = Bragg angle while in Bragg’s Law d is the d-spacing. Besides that, EDX method may be used to determine the types of elements present in a sample as well as their concentration. It is also commonly used to estimate the elemental makeup of composites. Moving forward, XPS is a surface-sensitive quantifiable spectroscopic technique which relies on the photoelectric consequence that can detect the elements present in a material (elemental composition) or on its surface, as well as their chemical states, and the overall electronic structure and density of the electronic states in the material. Figure 9 displays the XPS spectra of TiO_2_, GO and TiO_2_-GO composites. The TiO_2_ and TiO_2_-GO show two peaks at the binding energies of 458.5 and 464.3 eV for Ti 2p, which was assigned to the Ti 2p_1/2_ and Ti 2p_3/2_. The spectrum of TiO_2_ demonstrated two peaks of O 1s at 529.2 and 532.3 eV. The peaks of O 1s at about 530.3 and 531.7 eV were from the signal of O-H, C=O and C-O of GO. The TiO_2_ revealed four peaks at the binding energy of 529.2, 530.8, 532, and 533 eV, respectively, corresponding to the oxygen atoms of TiO_2_ and GO (Shi et al., 2021).

### Others

Thermal analysis of composites can be conducted through various instrumentation methods. Among all, thermogravimetric–differential thermal analysis (TG/DTA) is the most reported approaches used to illustrate the material’s thermal stability and thermal potency. Other than that, particle size analysis (PSA) is a method for determining the average size of nanocomposites. Meanwhile, Brunauer–Emmett–Teller (BET) analysis is also can be employed to study the specific surface area and porosity of composites.

## Graphene Oxide/Metal Oxide Nanocomposites as Photocatalyst

A number of researches were published recently concerning the use as photocatalysts of graphene/metal oxide nanocomposites for their outstanding qualities in water purification. These nanocomposites offer several advantages for example, increased conductivity, adjustable property, optical behavior, durability stability and lifespan. The GO was nanohybridized with a range of metal oxides, including ZnO, TiO_2_, WO_3_, Fe_2_O_3_, SnO_2_, CuO, etc. Graphene derivatives inhibit corrosion, and the liquidation into the water of metal oxide nanoparticles, other than restricting the recombination of the electron hole, thereby extending the photocatalytic lifetime (Upadhyay et al., 2014). The waste-derived synthesized graphene derivatives composite with metal oxide can bring a major breakthrough in the photocatalysis field.

### Graphene Derivatives/Zinc Oxide Nanocomposite as Photocatalyst

Researchers have spent a considerable effort over the past 2 decades in developing the photo-oxidation of organic dyes using GO-based ZnO nanocomposites. The development of heterostructures GO/ZnO and rGO-ZnO appears to reduce the recombinant losses and to extend the light response to visible light, which results in better photocatalytic functioning, particularly in visible light treatment (Atchudan et al., 2016). Table 1 highlights the GO/ZnO and rGO/ZnO nanocomposite photocatalysts for organic pollutants, especially for the technique of synthesis, shape of photocatalysts, as well as for the photocatalyst conditions and performance, described in literature. This section includes a number of intriguing nanocomposites GO/ZnO for organic dyes photo-oxidation.

**TABLE 1:** Confrontation of GO/ZnO and rGO/ZnO nanocomposite photocatalytic in the photo-oxidation of organic dyes with synthesis, and photo-catalytic performance.

Photocatalyst	ZnO morphology	Synthesis method	Targeted pollutant	Removal (%)	Ref.
GO/ZnO	Nanoparticles	Solvothermal	Congo Red	68	Atchudan et al. (2017)
GO/ZnO	Nanoparticles	Solvothermal	Methylene blue	98.5	Atchudan et al. (2016)
GO/ZnO	Nanoparticles	Solvent-free synthesis	Methylene blue	100	Lonkar et al. (2019)
GO/ZnO	Nanorod films	Hydrothermal	Methylene blue	99	Rokhsat and Akhavan, (2016)
GO/ZnO	Microspheres	Simple solution method	Methylene blue	99	Qin et al. (2017)
rGO/ZnO	Nanorods	Chemical etching and hydrolysis Method	Rhodamine B	92	Zhao et al. (2017)
rGO/ZnO	Nanorods	Hydrothermal	Methyl orange	93	Ranjith et al. (2017)
GO/ZnO	Nanoparticles	Simple solution approach	Safranin T	100	Nenavathu et al. (2018)
GO/ZnO	Nanoparticles	Solvothermal	Methylene blue	80	Jabeen et al. (2017)
GO/ZnO	Film	Atomic layer deposition	Methyl orange	84	Justh et al. (2018)
GO/ZnO	Nanoparticles	Sol-gel	Rhodamine B	99	Yao et al. (2016)
GO/ZnO	Nanoparticles	Ultrasonication + Hydrothermal	Methylene blue	99	Mohamed et al. (2019)
rGO/ZnO	Spindle	Hydrothermal	Methylene blue	93	Prabhu et al. (2018)
rGO/ZnO	Lotus	Sol-gel	Phenol	86	Yadav et al. (2019)
rGO/ZnO	Nanosheets	Hydrothermal	Methylene blue	100	Liu et al. (2020)
rGO/ZnO	Nanoparticles	Hydrothermal	Methylene blue	100	Van Tuan et al. (2020)
rGO/ZnO	Nanowires	Electrodeposition	Methylene blue	23	Pruna et al. (2017)

Posa et al. have synthesized GO/ZnO nanocomposites, utilizing a simple wet chemical approach for effective photo-mineralization under sunshine (Posa et al., 2016). Related to the development of the GO/ZnO, which increased the lifespan of transporters, and the excitation of the dyes as the visible light sensitizing, the extraordinary photocatalytic activity of the nanocomposites. It was due to the increased effectiveness of the load segregation of electric-hole carriers. The GO/ZnO nanocomposites were created by the integration of GO (i.e., 186.5 m^2^/g), which has more area than GO/ZnO nanocomposites, for photocatalysts with highly approachable surfaces (e.g., 158.0 m^2^/g). The GO/ZnO nanocomposites preparation through solvothermal method was reported by Atchudan et al. (Atchudan et al., 2016). Before solvothermal impregnation, the GO and ZnO nanoparticles were produced by Hummers’ methods and thermal oxidation. These combinations have been efficacious in photodegradation of methylene blue dye with UV light radiation, 98.5% after 15 min of irradiation. It was carried out due to the enhanced light absorption and decreased electron hole combination, which has been caused by the development of a GO/ZnO heterostructure. On the other hand, Qin et al. prepared the rGO/ZnO microspheres nanocomposite with an easy solution for the degeneration by UV light of Methylene Blue (Qin et al., 2017). The ZnO microspheres were morphological and composed of ZnO nanorods with uniform distribution of about 30 nm in diameter and roughly 150 nm in length. Compared to ZnO, the increased photocatalytic activity of ZnO-filling ZnO-rGO nanocomposites was due to the decrease in the recombinants process. Moreover, Liu et al. have manufactured rGO/ZnO nanocomposites based on well-circulated ZnO-nanocrystals on rGO using a simple microwave-assisted method in anhydrous medium (Y. Liu et al., 2012). In the decolorization of several dyes (methylene blue and rhodamine B), the produced nanocomposites showed better photocatalytic activity in visible light radiation. The authors predicted a considerable reduction in the process of recombination due to the creation of rGO/ZnO nanocomposites that can turnover considerably from photocatalytic efficiency of dyes (Yaqoob, Noor, et al., 2020).

### Graphene Derivatives/TiO_2_ Nanocomposite as Photocatalyst

TiO_2_ has been investigated for the treatment of water. It is one of the most popular and commonly utilized photocatalysts. It is preferable used as compared to other metal oxides due to its important properties such as safety, nontoxic, inexpensive, and chemically stable (Hunge et al., 2020). Similar to ZnO, its photocatalytic activity is solely confined to the ultra-violet region which is only 4% of the total solar energy and thus becoming a major problem (Yadav et al., 2021). This is due to the fact that TiO_2_ possess a wide bandgap energy (3.2 eV) (Koli et al., 2017; Delekar et al., 2018). A number of strategies have been explored to narrow the gap in the bands to make them active in an area of visibility such as doping, insertion of defects, and combination of these with electron acceptors. One of the most commonly researched of these techniques, along with electron acceptors. Graphene was also investigated as a molecule for electron acceptor composites with TiO_2_, and several composites with TiO_2_/graphene reported in different investigations are included in Table 2.

**TABLE 2:** Summary of graphene/TiO_2_ nanocomposite for photodegradation of water pollutants.

Photocatalyst	TiO_2_ morphology	Targeted pollutant	Removal (%)	Ref.
Graphene/TiO_2_ nanocomposite	Nanoparticles	Methylene blue	90	Cho et al. (2015)
Graphene/TiO_2_ nanocomposite	Nanoparticles	Methylene blue	100	Zhang et al. (2017)
Graphene/TiO_2_ composite	-	Methylene blue	85	Divya et al. (2017)
rGO/TiO_2_	Nanoparticles	Methylene blue	92	Sohail et al. (2017)
Graphene quantum dots/TiO_2_ nanocomposite	Nanoparticles	Methylene blue	100	Gupta et al. (2015)
rGO/TiO2	Anatase and nanofibers	Methyl orange	97	Lavanya et al. (2017)
Graphene-pasted-TiO2 composite	Spheres	Methyl orange	95	Xu et al. (2016)
GO/TiO_2_ nanocomposite	Nanoparticles	Methyl orange	88	Zhang et al. (2017)
rGO/TiO_2_ nanocomposite	Nanoparticles	Methyl orange	80	Zhang et al. (2017)
rGO/TiO_2_ nanocomposite	Nanotubes	Methyl orange	100	Zhao et al. (2015)
rGO/TiO_2_	-	Rhodamine B	100	Chen et al. (2017)
rGO/TiO_2_	Nanosheet	Rhodamine B	83	Wang et al. (2017)
rGO/TiO_2_-Au nanocomposite	Nanoparticles	Rhodamine B	100	Wang et al. (2017)
Core-shell TiO_2_/Graphene	-	Rhodamine B	100	Biris et al. (2016)
rGO/TiO_2_	Nanoflower	Rhodamine B	100	Kim et al. (2016)
rGO/TiO_2_	Nanotubes	Rhodamine B	100	Liu et al. (2016)
Graphene/TiO_2_	Nanoparticles	Methylene blue	100	Yang et al. (2016)
Graphene/TiO_2_	Nanoparticles	Methylene blue	100	Suave et al. (2017)
Graphite/TiO_2_	-	Methylene blue	100	Baldissarelli et al. (2015)
TiO_2_/Graphene	Flocculent	Methyl orange	70	Han et al. (2015)

A combination of nanoparticles of metal oxide and graphene derivatives, resulted in the formulation of new valence bands which is responsible to reduce the metal oxide band gap by hybridizing the atomic orbits. The amount of metal oxide loading on the graphic supporting material needs to be carefully adjusted for an effective catalyst-support interaction (Upadhyay et al., 2014). Indeed, in deciding on the photocatalytic activity of the combinations, the percentage of the graphene content plays a major role and changes in the quantity of graphene have a significant impact on the performance of the resultant catalyst. In general, the increase in composite graphene content increases photocatalysis, but an increase of graphene content above some threshold limit may lead to the decrease the photocatalytic activity by increasing the absorption and spread of photons by the excess carbon content in the composite. In order to achieve maximal photocatalytic activity, Wang et al. examined the impact of graph loading on TiO_2_–graphene composites and adjusted threshold weights for graphene (0.05 wt%) (Wang et al., 2012). The photocatalytic activity of the hybrid material intensely coupled TiO_2_ with graphene promotes load separation and delayed recombination. TiO_2_ can provide a significantly more effective photocatalyst for hybrid graphene or graphene *in situ* on TiO_2_. For TiO_2_/graphene-like carbon structure production, Wang et al. established a technique which shows over 2.5 times increased methylene blue dye photodegradation as compared with Pristine Degussa P25 TiO_2_ (Wang et al., 2010). Liang et al. described the development, using hydrolyze combined hydrothermal treatments, of TiO_2_ nanocrystals in a steady growth on the graphene oxide substrate (Liang et al., 2010). The GO/TiO_2_ hybrids were developed to demonstrate three-fold photocatalytic activity as compared to P25 TiO_2_ for the degradation of Rhodamine B dye. In another work, Min et al. effectively produced N doped TiO_2_ composites with graphene and examined their benzoic acid degradation photocatalytic activity (Min et al., 2013). The TiO_2_/graphene composites were doped with nitrogen and show increased photocatalytic activity as compared with pure TiO_2_-graphene composites that were subsequently ascribed to the increased N-TiO_2_/graphene composite response in the visible area. Additionally, TiO_2_/graphene composite surface modifiers may also increase the photocatalysis of the composite, using metal ions such as Pt, Ag, Fe, Au, etc. (Ahmad et al., 2021; Yaqoob et al., 2020). These metals are found to extend the life of charging carriers to capture excited electrons in order to decrease the recombination of charging carriers further.

### Nanocomposites of Graphene Derivatives With Other Metal Oxide as Photocatalyst

Other than TiO_2_ and ZnO, other metal oxide like WO_3_, Fe_2_O_3_, CuO and SnO_2_ are considered as nontoxic photocatalyst for pollutant removal due to their low-cost, fast response and recovery time. WO_3_, is an n-type semiconductor, activated under visible light irradiation having small bandgap (2.4–2.8 eV) that makes it a suitable catalyst for degrading organic compounds (Yadav et al., 2021). Further, Fe_2_O_3_ is another example of n-type semiconductor with narrow bandgap energy of 2.0–2.2 eV and it exhibits a wide photoelectrochemical response that is useful for photocatalysis (Botsa et al., 2020). In the case of CuO, it is a p-type semiconductor material with good optical and catalytic properties. It has a low bandgap energy of around 1.2–1.7 eV (Wang et al., 2020). On the other hand, SnO_2_ is an n-type semiconductor with a wide bandgap energy of 3.6 eV (Choudhari et al., 2020). It has a rutile crystal structure and very identical to TiO_2_. Due to the wide bandgap, SnO_2_ only active under UV light and very limited under the visible light (Shyamala and Gomathi Devi, 2020). Each type of these metal oxides has their own advantages and limitations. In some cases, combining them could minimize their limitation and subsequently enhanced their photocatalytic efficacy. Table 3 displays some examples of reported nanocomposites of graphene derivatives with other metal oxide with targeted pollutants and their removal performance.

**TABLE 3:** Summary of graphene derivatives with WO_3_ and Fe_2_O_3_ nanocomposites for photodegradation of dye pollutants.

Photocatalyst	Metal oxide morphology	Targeted pollutant	Removal (%)	Ref.
rGO/WO_3_ nanoplate	Nanoparticles	Congo red	94	Yadav et al. (2021)
rGO/WO_3_ nanocomposite	Nanoparticles	Methylene blue	82	Kodarkar et al. (2021)
rGO/WO_3_ nanocomposite	Nanorod	Rhodamine B	96	Govindaraj et al. (2021)
Indigo-rGO/WO_3_ nanocomposite	-	Methylene blue	80	Khan et al. (2019)
GO/WO_3_ nanocomposite	Nanorod	Methylene blue	83	Hu et al. (2018)
GO/α-Fe_2_O_3_ nanocomposite	Nanoparticles	Methylene blue	90	Mandal et al. (2018)
Graphene/α-Fe_2_O_3_	-	Rhodamine B	99	Frindy and Sillanpää, (2020)
Graphene/Fe_2_O_3_/CuO	-	Methylene blue	94	Nuengmatcha et al. (2019)
Graphene/Fe_2_O_3_/TiO_2_ nanocomposite	-	Rhodamine B	97	Zhang et al. (2018)
GO/Fe_2_O_3_/SnO_2_ nanocomposite	-	Methylene blue	98	Botsa et al. (2020)
rGO/SnO_2_	Aerogel	Methyl orange	84	Kim et al. (2019)
rGO-SnO2	-	Rhodamine B	98	Kim et al. (2019)

## Mechanism of Photocatalytic Degradation

**FIGURE 10:**
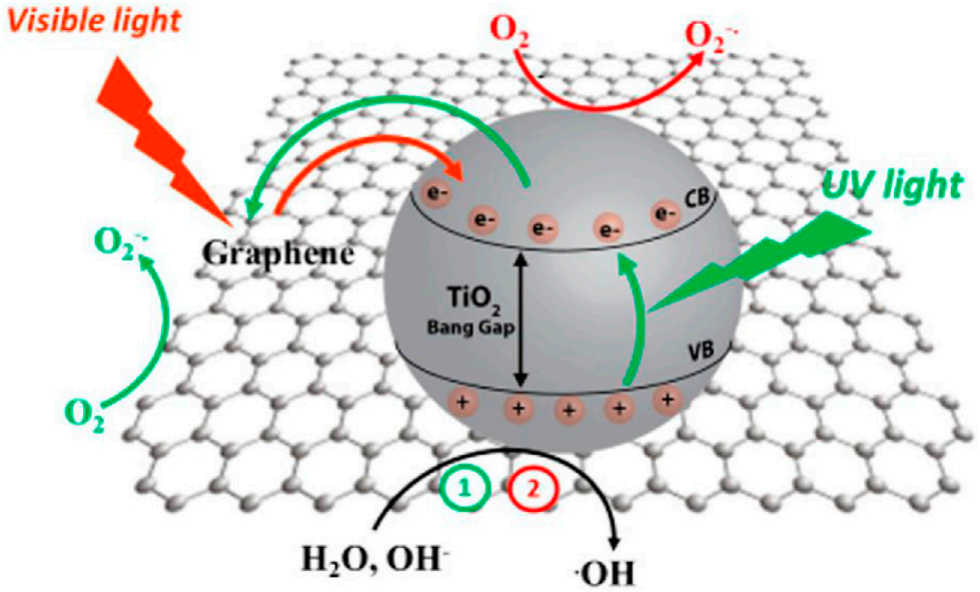
Mechanism of photodegradation by using graphene/TiO_2_ as a photocatalyst in the presence of visible light (Adapted from Giovannetti et al. (2017) with MDPI permission).

As far as the mechanism of photocatalytic degradation is concern, the radical species that generated in the semiconductor photoexcitation played an important role for organic pollutant’s degradation. The pictorial illustration of degradation using graphene/TiO_2_ as a photocatalyst in the presence of visible light is presented in Figure 10.

The most crucial steps concerned in this degradation process can be pictured in the steps, are mentioned below (Eqs. 5–11) (Qi et al., 2017).

$$\mathrm{Photons}\;\left(hv\right)+\mathrm{semiconductor}\to h^{+}\left(VB\right)+e^{-}\left(CB\right)$$ (5)

$$h^{+}+H_{2}O\to H^{+}+OH^{-}$$ (6)

$$h^{+}+OH^{-}\to {\mathrm{OH}}^{•}$$ (7)

$$e^{-}+O_{2}\to O_{2}^{•}$$ (8)

$${2\text{e}}^{-}{+\text{O}}_{2}{+\text{H}}_{2}\text{O}\to H_{2}O_{2}$$ (9)

$$e^{-}+H_{2}O_{2}\to {\mathrm{OH}}^{•}+\;OH^{\_}$$ (10)

$$\mathrm{Organic}\;contaminant+OH^{•}+O_{2}\to CO_{2}+H_{2}O+\mathrm{other}\;degraded\;products$$ (11)

The mechanism also depends on certain conditions of experiment. The degradation mostly depends on the excited electron with hole inside the semiconductor. A variety of semiconductors are being employed with mostly nanosized state owing to increases of surface area and effect of quantum size favorably (Samadi et al., 2019). Different forms of ZnO or TiO_2_ with either metal or non-metal doping can be applied in the various types of pollutant degradation due to stability, non-toxic properties and capability in degradation of ZnO and TiO_2_ (Lum et al., 2020). Nevertheless, the application of this compound has been restricted for the degradation of organic pollutant commercially because of the possible variables related to experiment including, light used and separation treatment technique. The TiO_2_ was the most popular photocatalyst material in the early days. However, nowadays, more research at a higher level has been required in order to explore better alternatives rather than ${\text{TiO}}_{2}$ in photocatalytic degradation application. Another compound can be better employed than ${\text{TiO}}_{2}$ such as ZnO and their composite-based material (Gebreslassie et al., 2013). The reasonable reason for this choice is believed to be the ${\text{TiO}}_{2}$ high absorption coefficient. The intensity of semiconductor scattering on the surface can vary and thus, the most variable is more accounted for the roughness factor value on the surfaces. Furthermore, the absorption coefficient value depends on different physical features such as roughness of surface, the size particle of inherent valence electrons occupancy added and the occupied wave functions. However, ZnO and TiO_2_ can be an ideal approach for photocatalysis in the form of composites with other materials.

## Factor Affecting the Photocatalysis Process

There are several factors which hold direct effects on the performance of photocatalysis to degrade/decolorize the pollutants from water resources. Some of the most important are mentioned here to show their significance in photocatalysis.

### Photocatalyst Loading

The photocatalyst loading effect in particular ZnO or TiO_2_ doped carries a substantial effect on performance of photocatalytic behaviour. In literature, different photocatalyst studies have been already investigated to observe their loading effect on the entire performance of pollutant’s degradation (Zhang et al., 2017). After extensive literature review, the conclusion demonstrated that the photodegradation is relative to the loaded catalyst until it achieved an elevated condition. This phenomenon corresponds to the high photocatalyst quantity which raises the entire active reaction spots and ZnO/GO photocatalyst surface area (Rajamanickam and Shanthi, 2016; Vaiano et al., 2018). As an effect, the amount of superoxide radicals and hydroxyl increased which enables the degradation of pollutants. Therefore, the percentage of degradation was improved. Though, the photodegradation percentage reduces at high concentration loadings of the catalyst when the dose was at optimized concentration due to screening effects and UV-light scattering influence. Furthermore, a high amount of photocatalyst promotes the agglomeration contact which may lead to reduced catalytic surface area. This surface area is accessible for UV absorption and chemical adsorption, which ultimately decreased the catalytic effectiveness. One more effect observed is solution’s turbidity which also increases as a result of high catalyst loading. This singularity prevents the dispersion of light into mediums. Therefore, the photo-activated suspension volume reduces and thus degradation rate is lower respectively (Ng and Cheng, 2016; Lonkar et al., 2019). This phenomena recommended that the optimized photocatalyst mass must be investigated to avoid excessive dose of catalyst and certify a supreme photons absorption.

### Structure of Photocatalyst

The photodegradation efficiency can be improved by the modification in the development of ZnO and TiO_2_ structure. Recently, ZnO or TiO_2_ nano-based structure has received an excessive attention in photocatalysis study due to the presence of excellent physicochemical properties as well as structural morphology (Duan et al., 2020; Mohd Adnan et al., 2020; Zouhier et al., 2020). The ZnO or TiO_2_ NPs is available in several morphologies like nanosheets, nanobelts, nanorods, nano dumb bells, nanowires, nanospiral disks, neotheropods and nanoflowers. These all forms are different in properties and morphologies than bulk ZnO or TiO_2_ material. The ZnO and TiO_2_ nanostructured morphologies contain a nano range particle size with high surface area. This high surface area offers excellent physical and chemical properties (Giovannetti et al., 2017; Lum et al., 2020; Zhang et al., 2017). The nano range ZnO/TiO_2_ holds a unique surface and quantum effect. Assi et al. studied the ZnO nanorod for methylene blue (MB) degradation via photocatalysis (Assi et al., 2015). The study specified that the high surface area of ZnO nanorods produces improved photodegradation productivity than nanorods with low surface area. An innovative ZnO or TiO_2_ nano range sheet was created for the Rhodamine B photodegradation via photocatalysis process. However, the 3D fleecy-based structure proposes several reaction active sites and large surface area for operation (Lai et al., 2010). The Rhodamine B removal by photocatalytic reaction showed a higher rate of photodegradation than many other nanostructures catalysts due to presence of optimized surface area and enhanced isolation competence for holes and electrons.

### Oxidizing Species

The excellent oxidizing species addition, such as potassium peroxydisulfate (K_2_S_2_O_8_) and hydrogen peroxide (H_2_O_2_) into suspension of ZnO/TiO_2_ is common procedure used to increase the photo-oxidation rate (Wahab et al., 2019). Besides that, it is assumed that hydrogen peroxide has multifunction during the photocatalytic degradation process. For examples, it can draw electron away from conduction band which lead towards separation of charge promotion and hydrogen peroxide also generate hydroxyl radicals as shown below in Eqs. 12, 13.

$$H_{2}O_{2}+e^{-}\to {\mathrm{OH}}^{-}+OH^{•}$$ (12)

$$H_{2}O_{2}+O_{2}^{-}\to {\mathrm{OH}}^{-}+OH^{•}+O_{2}$$ (13)

When concentration of hydrogen peroxide is higher than concentration critical, it may act like hole or else scavenger for OH^**−**^ or become reactive towards ZnO/TiO_2_ in forming peroxyl groups that are unfavourable to photocatalytic reaction (Pathak et al., 2017). Bizani et al. stated in their report that oxidant addition like H_2_O_2_ proved to become more effective in the degradation of pollutants rather than K_2_S_2_O_8_ experimentally, even though their efficiency in degradation could be vice versa. Moreover, intermediates of organic pollutants had been shown to possess more toxicity when using K_2_S_2_O_8_ rather than degradation of organic pollutants by photocatalysis technique. It involves H_2_O_2_, which indicates efficiency of toxic removal and partial dissolved organic carbon (DOC) (Bizani et al., 2016).

### pH

The pH level is another key factor determining the degradation of organic compounds during photocatalytic reaction. In essence, pH has a substantial influence on the surface charge of catalyst, contaminant hydrolysis, oxidant and contaminant ionization degree (Hayati et al., 2018). The surface charge of the photocatalyst affected the adsorption properties of the cationic or anionic organic pollutant. Additionally, the pH of the solution influences the dominant oxidation species, whereby at lower and higher pH levels, holes and hydroxyl radicals were shown to be the most important oxidative species (Alamelu and Jaffar Ali, 2020). Another important parameter to comprehend is the point of zero charge (pzc); is a state in which the net surface charges of a catalyst is zero or neutral and falls within a specific pH level (Alkaim et al., 2014). The pzc determined the surface charge of catalysts, at which it is positively charge when the pH level is less than pzc and negatively charge beyond pzc (Yaqoob et al., 2020). Thus, finding the optimum pH condition is a crucial stage that could determine the degradation effectiveness of the catalysts.

### Temperature

The photocatalysis degradation development may lead to functions at optimized temperature as well as distinctive pressure because the photonic activation carries direct effect on it. This is valuable for water sanitization behaviour and the thermal phase, can be omitted to preserve power (Lee et al., 2016). Overall, high temperatures of reaction may improve the degradation rate of pollutants. Furthermore, it can decrease the adsorptive volumes of the reactant and liquified oxygen, it referred to the low photodegradation productivity (Kumar et al., 2020; Lum et al., 2020). So, an optimized condition is very necessary for better efficiency in presence of ZnO/GO and TiO_2_/GO composites as photocatalysts.

### Light Wavelength

The light wavelength also carries a straight effect on the performance of the photocatalysis process. In case of UV irradiation, it refers to the electromagnetic band which can be divided as UV-A1, UV-A2 and UV-A3 corresponding to its producing wavelength (Wang et al., 2013). The range of UV-A1 is within 315–400 nm or 3.10–3.94 eV while UV-A2 and UV-A3 have a range 280–315 nm and 100–280 nm respectively. However, studies showed that the UV-A1 light offers enough light photons of synthesized TiO_2_ for 1,8-diazabicyclo (5.4.0) undec-7-ene photodegradation (Lee et al., 2016). Photocatalyst productivity is often high at 254 nm because of lower dispersion capacities of high energy photons, which enhances the electron-hole pairs that are created accordingly for hazardous pollutant decompositions (Samadi et al., 2016).

## Studies of Intermediate Products Formed During Photocatalytic Degradation of Organic Pollutants

During degradation, organic pollutants are decomposed or degraded into a number of products, whereas these pollutants are completely destroyed into water, carbon dioxide and certain inorganic ions when mineralization is carried out. The HPLC-MS or GC-MS was applied for analysis of degraded products following pollutant degradation by using the nanomaterials (TiO_2_ or ZnO) or graphene nanocomposites material in the presence of light. In this case, it is required to establish a relationship between parent compound and intermediates, weather these products are more harmful or less harmful than parent compounds utilizing the toxicity test in respect of the degradation products acquired (Yaqoob et al., 2020). Moreover, the idea of mineralization is alike with complete photodegradation which determines the formation of CO_2_, H_2_O and inorganic salts during the degradation of a pollutant (Horikoshi et al., 2004; Hu et al., 2006). Other minerals also may include such as sulphide, ammonia, sulfite, fluoride, sulphide, chlorine, phosphate, nitrite, etc. during mineralization of compounds (Rasolevandi et al., 2019). In general, as a result of generating a stable intermediate in process, the rate of mineralization is lower compared with degradation. A long irradiation for total organic carbon (TOC) elimination is thus expected to be required. Furthermore, in order to breakdown organic substances, the mineralization idea avoids generating unwanted products (Celine et al., 2017; Kim et al., 2017). The TOC is determined by an organic substance and quantified by a TOC analyzer as the total quantity of confined carbons coupled with a photodegradation mechanism, show the probable method of pollutant mineralization.

**FIGURE 11:**
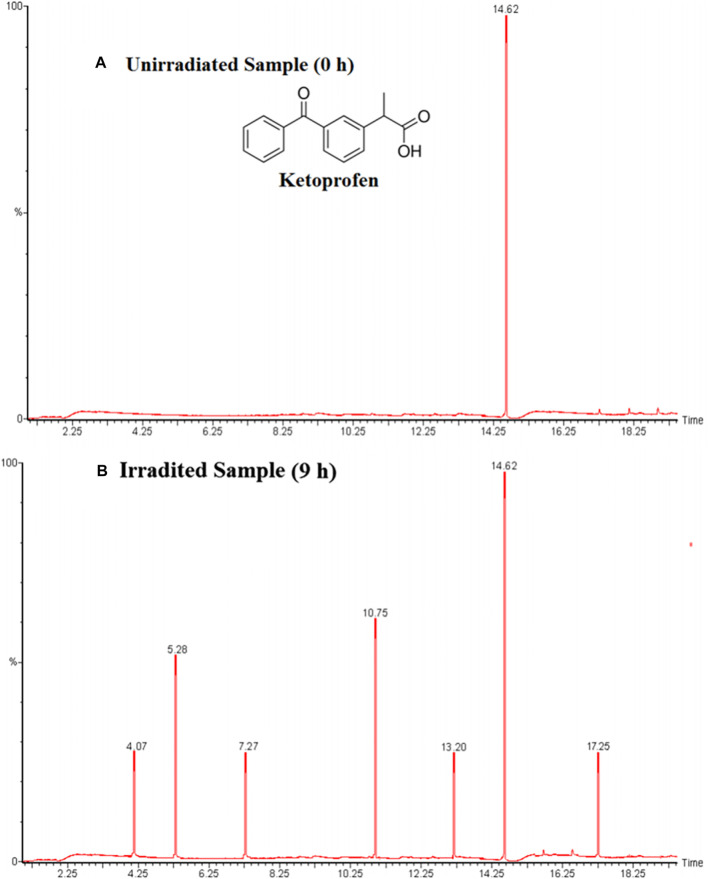
Ketoprofen gas chromatogram analysis **(A)** unirradiated mixture (0 h), **(B)** irradiated mixture (9 h). (Adapted from Umar et al. (2019) with Springer permission).

**FIGURE 12:**
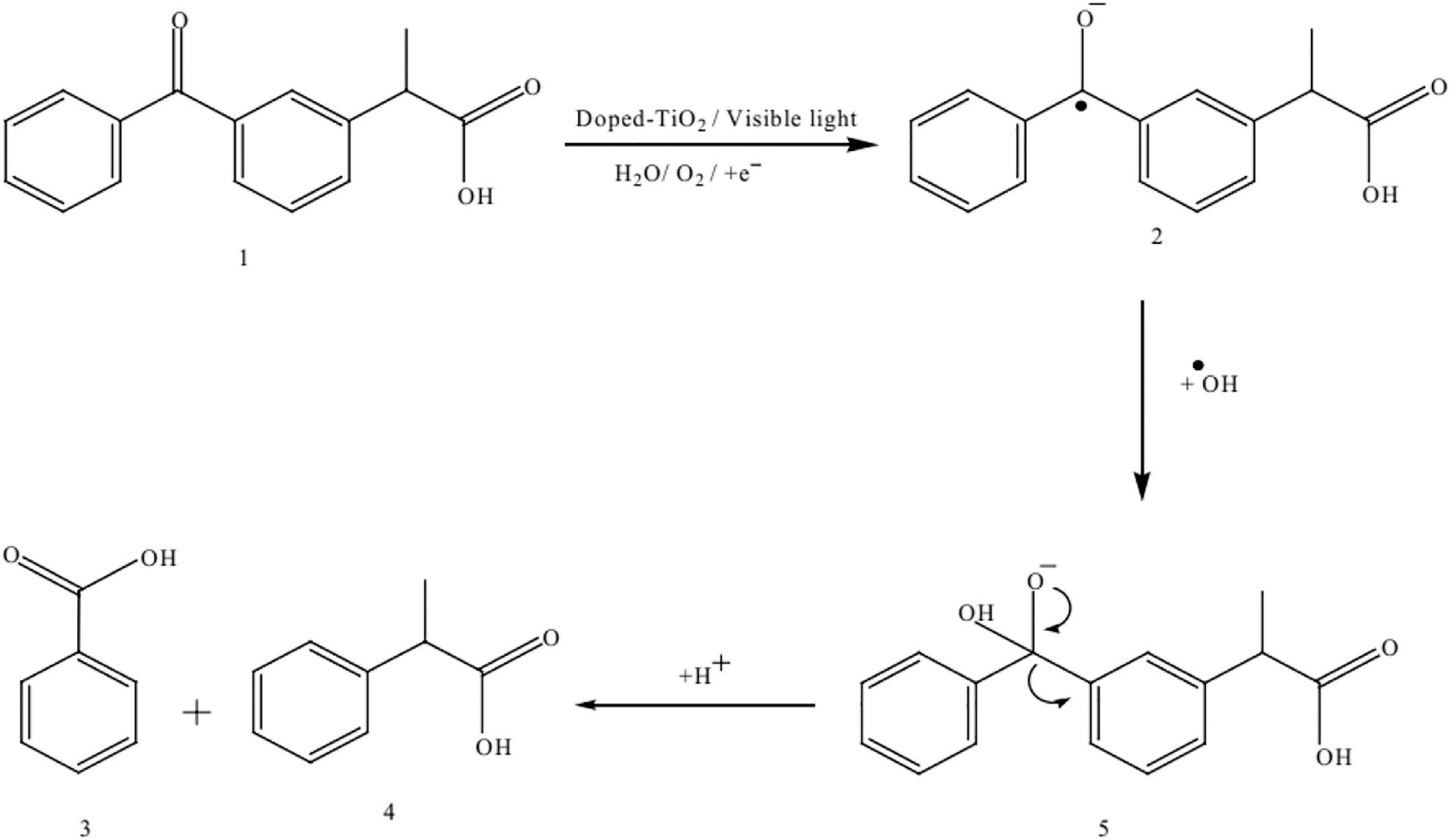
The photocatalytic degradation mechanism of ketoprofen by using the Mn-doped TiO_2_ as photocatalyst (Adapted from Umar et al. (2019) with Springer permission).

Few examples which are previously discussed in literature such as Umar et al. studied the ketoprofen and chlorothalonil photodegradation pathway and found intermediate products by using the Mn-doped TiO_2_ as a photocatalyst. Both analysis were analyzed using the GC–MS analytical techniques to detect the transitional outcomes produced during photooxidation (Umar et al., 2019). For example, a linear light-halogen lamp (500 W, 9,500 lumens) was used to irradiate the aqueous ketoprofen’s solution (0.2 mM) in the presence of Mn-doping TiO_2_ (3 g L^−1^). The GC-MS analysis of an unirradiated and irradiated Ketoprofen solution showed that a single peak (Rt = 14,62) was obtained in first case and several peaks were appeared at retention times (Rt) 4.07, 5.28, 7.27, 10.75, 13.20 and 17.25 in later case as shown in Figures 7B, 11A, respectively. Based on the a pattern of mass fragments and molecular ions, two products at Rt = 5.28 and 10.75-min are described by such as benzoic acid **(3)** and 2-phenylpropanoic acid **(4)** and a possible route for the formation of these products can be seen in Figure 12.

**FIGURE 13:**
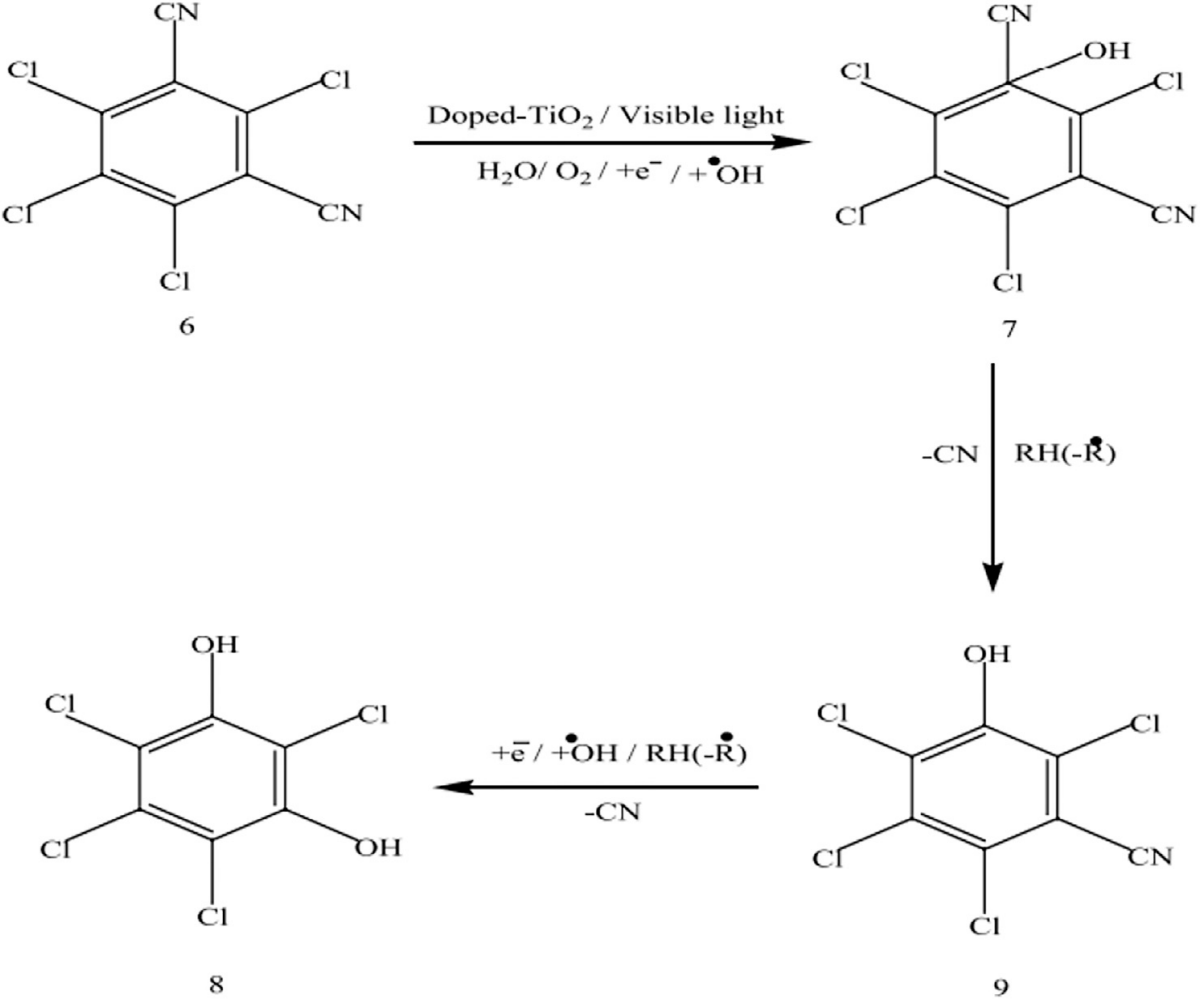
The photocatalytic degradation mechanism of chlorothalonil by using the Mn-doped TiO_2_ as photocatalyst (Adapted from Umar et al. (2019) with Springer permission).

Similarly, in the case of chlorothalonil, photodegradation and intermediate products are shown in Figure 13. Analysis of the 9 h irradiated chlorothalonil mixture indicates the generation of a number of intermediate yields, two of which appear to be *R*_*t*_ = 14.15 and 17.01 based on the pattern of mass fragmentation and molecular ions, were characterized as 4 and 3, which indicate a replacement of a cyanide group with a hydroxylated benzene ring group.

Similarly, the Basnet et al., studied the Mn-ZnO composite as nano-photocatalyst in order to degrade the methylene blue (MB) dye solution (Basnet et al., 2021). The ESI-MS analyses examined the degradation process for MB solution. The spectrums ESI-MS obtained in 1) 0 min 2) 10 min (15 min) 4) 20 min and the first MB solution analysis of ESI-MS at 0 min shows the existence of molecular ion (M^+^) peak at m/Z = 284. The peaks of m/Z of 304–306, can be attributable to the center of MB, generated by a central aromatic heterocyclic aperture by the electromagnetic interaction between •OH and sulfhydryl (C -S^+^ = C), leading to sulfoxide moiety production (C—S(=O)—C) (Trandafilović et al., 2017; Yang et al., 2017). As a consequence of the contamination by Azure B and Azure A, as a result of partial de-methylating products of MB, peaks with m/Z values of 270 and 256 appeared (Depuccio et al., 2015). Low-intensity peaks in m/Z = 272, 253, 229, 217, 149, 129, 110 after 10 min of light irradiation suggest breakup of molecules of MB (Molla et al., 2015). MB solution ESI-MS analysis. These intermediates have also dissolved into many tiny intermediate molecular masses, which have eventually become CO_2_, H_2_O, and inorganic salts (Gnaser et al., 2005). Based on this investigation, the aqueous MB dye solution has been suggested as a viable photocatalytic degradation route.

Furthermore, Rajamanickam et al. studied the degradation of 4-nitrophenol by using the ZnO as photocatalyst (Rajamanickam and Shanthi, 2016). The formed intermediate products during the photocatalytic activities were detected by GC–MS analysis. Organic pollutant photodegradation processes can be carried out by the formation of hazardous intermediates which are poisonous greater than the initial molecules. Therefore, in photocatalytic degradation processes, it is essential to determine the identities of intermediates. In the photocatalytic degradation of 4-nitrophenol, an attempt has been made to find the intermediate intermediates forming in the GC-MS analysis. After 30- and 60-min irradiation, GC–MS analysis of the solution was done. For retention periods of 18.0, 21.6, 27.9 and 29.4 min, it was found four prevalent peaks. Eight products have been identified on the basis of their molecular ions and peaks for mass spectrometry.

Similarly, Cao et al., studied the routes for the formation of intermediate products (tetrabromobisphenol (TBBPA)-A) using the graphene–TiO_2_ composite as a photocatalyst. LC–MS was used to examine TBBPA intermediates in the photocatalytic degradation process. The photocatalytic degradation route of TBBPA was postulated based on the identified intermediates. The TBBPA decomposition was nearly 100% within 60 min with the formation of several intermediates, which were discovered by mass spectrometry. The mass spectra peak detected five major production ions at 465 m/z, 401 m/z. 387 m/z, 307 m/z and 227 m/z, which were labeled Products A–E as presented in Table 4 (Cao et al., 2015).

**TABLE 4:** TBBPA and its potential intermediates were found during the photodegradation process by graphene–TiO_2_ in the presence of UV light.

Product	Compounds	Retention time/min	m/z
	TBBPA	1.625	544
A	tri-BBPA	1.433	465
B1	4-(2-(3,5-dibromo-4-hydroxyphenyl)propan-2-yl)benzene-1,2-diol	6.979	401
B2	3-bromo-5-(2-(3-b romo-4-hydroxyphenyl)propan-2-yl)benzene-1,2-diol	6.979	401
C1	di-BBPA	1.475	386
C2	di-BBPA	1.475	386
D	mono-BBPA	1.634	307
E	BPA	23.182	227

By/ comparing the mass spectra with identified products from the degradation of TBBPA reported in previous published reports (Liu et al., 2013; Luo et al., 2010). The products A, C–E were identified as tribromobisphenol A (tri-BBPA), dibromobisphenol A (di-BBPA, two isomers), monobromobisphenol A (mono-BBPA), and bisphenol A (BPA), respectively. Product B was discovered as isomers of 4-(2-(3,5-dibromo-4-hydroxyphenyl) propan-2-yl) benzene-1,2-diol (B1) and 3-bromo-5-(2-(3-bromo-4-hydroxyphenyl) propan-2-yl) benzene-1,2-diol (B2). The presence of the other intermediates was not found in this study, which is likely owing to their low concentration. The relative peak area of TBBPA and these intermediates was compared to determine their concentrations, as illustrated in Cao et al., article (Cao et al., 2015). The peak area of TBBPA vanished after 60 min, which was consistent with the HPLC findings.

Furthermore, the peak area of the above-mentioned intermediates increased, implying the emergence of additional substrates, namely intermediates and products. The peak areas of product A and product D both increased initially and then declined, indicating that their appearance was dominating in the first stage. Then both Product A and Product D were rapidly degraded. After 30 and 10 min, respectively, the peak areas of Product B1/B2 and Product E rose rapidly and reached a maximum. Then, when the irradiation duration was extended, they altered irregularly. Product E was not the end product, as can be observed, and it may have been converted into some tiny organic compounds that were not discovered in our investigation. On the other hand, over the entire 60 min, the peak area of Product C1/C2 rose steadily and constantly. It was discovered that Product C1/C2 was most likely stable and/or that their creation was faster than their degradation during the procedure. Further, the potential mechanism for the photodegradation of TBBPA by graphene–TiO_2_ composites was postulated based on the preceding discussion of identified intermediates and prior studies. It was also noticed that TBBPA was photodegraded by three different pathways: debromination, substitution, and dihydroxylation. However, according to an extensive literature survey there were not many studies on discussion of graphene derivatives with ZnO or TiO_2_ nanocomposite photodegradation by using GC–MS or EIS-MS. Few studies of ZnO or TiO_2_ were characterized by GC–MS or EIS-MS.

## Present Challenges and Future Perspectives

To date, a wide variety of nanocomposites from graphene and its derivative with metal oxides are being developed for use in photocatalysis field. The research in this area is still ongoing and continually expanding as they have to overcome the technological and economic barriers in order to be practically viable. There are several concerns about the existing systems that must be addressed. Firstly, there are presently no ways of producing graphene derivatives in huge amounts which are scalable and affordable despite their remarkable applicability. Secondly, researchers have demonstrated that the photocatalytic performance of these graphene-based nanocomposites could be increase by enhancing the absorption of light, load separation and conveyance, and a longer operational life of the nanocomposite systems. Based on this point of view, some constrain need to be focused on to overcome the major scientific and technical problems preventing their utilization in applications at large scale and make it unsuitable at economic importance. For example the synthesis and design of the new graphene-based nanocomposites for photocatalyst still require integral thinking; hence the success of photocatalysis will demand future interdisciplinary effort between researchers and engineers. This approach might be useful in addressing the post-revival, recyclability, and reusability of nanocomposites. Next is the stability in the development of photocatalysts is another significant problem due to their limited useful lives and photocorrosion. For example, ZnO or TiO_2_-based photocatalysts, photocorrosion is more critical than chemical corrosion. To date, numerous researches have concentrated on the improvement of ZnO or TiO_2_-based photocatalyst photocorrosion resistance and this should be extended to other system too. Further, bioinspiration can be a suitable technique to build new, effective, light-trapped, pollutant-absorbing designs. The synthesis of GO from waste material is an ideal approach to overcome the synthesis problem.

## Conclusion

In summary, graphene/ZnO and graphene/TiO_2_ nanocomposites were studied in the expansion of new and futuristic photocatalysis materials. The present review highlighted the literature survey about graphene/ZnO and graphene/TiO_2_ nanocomposites from 2010 to 2020. The coupling between graphene and photoactive metal oxide semiconductors increases the photocatalytic function of metal oxide for the destruction of different aquatic contaminants such as organic dyes, heavy metal ions and pathogens. Graphene’s unique features in conjunction with nanomaterials’ size-dependent features provide other capabilities such as high adsorption capacity, wider light absorption range and better charging separation capabilities and great stability. The development of graphene/metal oxide nanocomposite-based water treatment technology is a very intriguing but tough challenge for researchers since there still are few issues, such as what the future of these composites will be like, and how environmentally friendly do they become? This also represents an important problem for the recycling and disposal of these expired composites. Strategies for the recycling and disposal in home water purity devices for waste creation will be developed concurrently. Finally, the conclusion is, the graphene/metal oxide nanocomposite has enormous possibilities as an effective photocatalyst, the problems with these materials need to be solved, for example with the nature of the chemical interaction or the bonding of metal oxides and the graphene. The need to study them carefully in order to make a more efficient photocatalyst is not fully known. In order to maximize output from these composites, thorough research on the optimization of the graphene content in the composite is needed.
